# PReS-FINAL-2265: Tuberculosis in pediatric patients who are receiving anti-TNF agents

**DOI:** 10.1186/1546-0096-11-S2-P255

**Published:** 2013-12-05

**Authors:** J Calzada-Hernández, A Noguera Julian, S Ricart Campos, R Bou Torrent, E Iglesias Jiménez, MI González Fernández, J Sánchez Manubens, V Torrente Segarra, L Rozas Quesada, FJ Martín Carpi, J Antón López

**Affiliations:** 1 Pediatric Rheumatology Unit, Pediatrics Department, Hospital Sant Joan de Déu, Esplugues de Llobregat (Barcelona), Spain; 2 Pediatric Infectious Diseases Unit, Pediatrics Department, Hospital Sant Joan de Déu, Esplugues de Llobregat (Barcelona), Spain; 3 Pediatric Gastroenterology Department, Hospital Sant Joan de Déu, Esplugues de Llobregat (Barcelona), Spain

## Introduction

Adult patients receiving anti-TNFα treatment are at increased risk for developing tuberculosis (TB). Few data have been published in the pediatric population.

## Objectives

We describe the occurrence of latent tuberculosis infection (LTI) and TB in children and adolescents treated with anti-TNFα agents.

## Methods

Cohort observational study including pediatric patients receiving anti-TNFα agents in a tertiary-care pediatric hospital. LTI is ruled out by the implementation of anti-TNFα drugs by tuberculin skin test (TST) and, from March 2012, QuantiFERON Gold-In Tube^® ^test (QTF). Along treatment, patients are evaluated periodically for TB using history and physical examination, but TST/QTF are not systematically repeated.

## Results

The final cohort consisted of 261 anti-TNFα treatments in 221 patients (56.1% female), of whom 51.7%/31.%/17.2% treated with etanercept/adalimumab/infliximab, respectively, for a variety of rheumatic diseases (75.6%), inflammatory bowel disease (20.8%) and inflammatory eye diseases (3.6%). The mean(SD) age at diagnosis of the primary condition was 7.2(4.6) years and the duration of the disease before implementing the anti-TNFα agent was 3.0(3.3) years. The total follow-up time under anti-TNFα treatment was 614 patients-year; mean(SD) time per patient: 2.8(2.2) years.

LTI was diagnosed in 3 adolescent girls (prevalence rate: 1.4%; 95%CI: 0-2.9) affected with juvenile idiopathic arthritis, who received isoniazid chemoprophylaxis and were later treated with anti-TNFα, without incidences. QTF tested positive in all three patients, while TST was positive in only one of them. No incident cases of TB were observed.

## Conclusion

In our study, the prevalence of LTI (1.4%) was similar to that reported in population screening studies in Spain and no incident cases of TB were observed.

## Disclosure of interest

None declared.

